# Efficacy and auditory biomarker analysis of fronto-temporal transcranial direct current stimulation (tDCS) in targeting cognitive impairment associated with recent-onset schizophrenia: study protocol for a multicenter randomized double-blind sham-controlled trial

**DOI:** 10.1186/s13063-023-07160-z

**Published:** 2023-02-24

**Authors:** Clément Dondé, Julien Bastin, Arnaud Pouchon, Nicolas Costes, Eric Fakra, Filipe Galvão, Aurélia Gay, Frédéric Haesebaert, Laurent Lamalle, Inès Mérida, Maxence Rigon, Fabien Schneider, Irène Troprès, Jérôme Brunelin, Mircea Polosan

**Affiliations:** 1 Univ. Grenoble Alpes, Inserm, CHU Grenoble Alpes, Grenoble Institut Neurosciences, 38000 Grenoble, France; 2 Adult Psychiatry Department CHU Grenoble Alpes, 38000 Grenoble, France; 3 Early Intervention Psychiatry Department, CH Alpes-Isère, F-38000 Saint-Egrève, France; 4 CERMEP-Imagerie du vivant, Lyon, France; 5 Psychiatry Department, University Hospital Saint-Etienne. INSERM, U1028; CNRS, UMR5292; Lyon Neuroscience Research Center, PSYR2 Team, F-69000 Lyon, France; 6 Centre Hospitalier le Vinatier, F-69500 Bron, France; 7 CHU Saint-Étienne, University Department of Psychiatry and Addiction, 42055 Saint-Étienne Cedex 2, France; 8 TAPE Laboratory, EA7423, Jean Monnet University, Saint-Étienne, France; 9 SUR-CL3R-PEPS, Centre Hospitalier Le Vinatier, PSYR2 team, Bat 416 – 1st floor; 95 boulevard Pinel, 69678, F-69500 Bron cedex, France; 10 INSERM, U1028; CNRS, UMR5292, Lyon Neuroscience Research Center, PSYR2 Team, F-69000 Lyon, France; 11 Lyon 1 University, F-69000 Villeurbanne, France; 12 Univ. Grenoble Alpes, UMS IRMaGe CHU Grenoble, 38000 Grenoble, France; 13 Psychiatry Department, University Hospital Saint-Etienne, Saint Etienne, France; 14 Service de Radiologie, CHU de Saint Etienne TAPE EA 7423, Université Jean Monnet, Saint Etienne, France; 15 Centre Hospitalier Le Vinatier, PSYR2 team, Bat 416 – 1st floor; 95 boulevard Pinel, 69678, F-69500 Bron cedex, France; 16 INSERM, U1028; CNRS, UMR5292; Lyon Neuroscience Research Center, PSYR2 Team, F-69000 Lyon, France; 17 Lyon 1 University, F-69000 Villeurbanne, France; 18 Université Jean Monnet Saint Etienne, F-42000 Saint Etienne, France

**Keywords:** Psychiatry, Schizophrenia, RCT, Noninvasive brain stimulation, tDCS, Cognitive impairment, Early auditory processing, Biomarker

## Abstract

**Background:**

In parallel to the traditional symptomatology, deficits in cognition (memory, attention, reasoning, social functioning) contribute significantly to disability and suffering in individuals with schizophrenia. Cognitive deficits have been closely linked to alterations in early auditory processes (EAP) that occur in auditory cortical areas. Preliminary evidence indicates that cognitive deficits in schizophrenia can be improved with a reliable and safe non-invasive brain stimulation technique called tDCS (transcranial direct current stimulation). However, a significant proportion of patients derive no cognitive benefits after tDCS treatment. Furthermore, the neurobiological mechanisms of cognitive changes after tDCS have been poorly explored in trials and are thus still unclear.

**Method:**

The study is designed as a randomized, double-blind, 2-arm parallel-group, sham-controlled, multicenter trial. Sixty participants with recent-onset schizophrenia and cognitive impairment will be randomly allocated to receive either active (*n*=30) or sham (*n*=30) tDCS (20-min, 2-mA, 10 sessions during 5 consecutive weekdays). The anode will be placed over the left dorsolateral prefrontal cortex and the cathode over the left auditory cortex. Cognition, tolerance, symptoms, general outcome and EAP (measured with EEG and multimodal MRI) will be assessed prior to tDCS (baseline), after the 10 sessions, and at 1- and 3-month follow-up. The primary outcome will be the number of responders, defined as participants demonstrating a cognitive improvement ≥*Z*=0.5 from baseline on the MATRICS Consensus Cognitive Battery total score at 1-month follow-up. Additionally, we will measure how differences in EAP modulate individual cognitive benefits from active tDCS and whether there are changes in EAP measures in responders after active tDCS.

**Discussion:**

Besides proposing a new fronto-temporal tDCS protocol by targeting the auditory cortical areas, we aim to conduct a randomized controlled trial (RCT) with follow-up assessments up to 3 months. In addition, this study will allow identifying and assessing the value of a wide range of neurobiological EAP measures for predicting and explaining cognitive deficit improvement after tDCS. The results of this trial will constitute a step toward the use of tDCS as a therapeutic tool for the treatment of cognitive impairment in recent-onset schizophrenia.

**Trial registration:**

ClinicalTrials.gov NCT05440955. Prospectively registered on July 1^st^, 2022.

**Supplementary Information:**

The online version contains supplementary material available at 10.1186/s13063-023-07160-z.

## Administrative information

Note: the numbers in curly brackets in this protocol refer to SPIRIT checklist item numbers. The order of the items has been modified to group similar items (see http://www.equator-network.org/reporting-guidelines/spirit-2013-statement-defining-standard-protocol-items-for-clinical-trials/).

**Table:** 

Title {1}	Efficacy and auditory biomarker analysis of fronto-temporal transcranial direct current stimulation (tDCS) in cognitive impairment associated with recent-onset schizophrenia: study protocol for a multicenter randomized double-blind sham-controlled trial *Short title:* tDCS for Cognitive Impairment Associated With Recent-onset Schizophrenia (STICOG) *Original French title:* Efficacité et tolérance de la stimulation électrique transcrânienne fronto-temporale gauche à courant continu (tDCS) comme traitement du déficit cognitif chez les sujets atteints de schizophrénie débutante : un essai multicentrique, randomisé, contrôlé.
Trial registration {2a and 2b}	Clinicaltrials.gov registration number: NCT05440955, first posted on July 1^st^, 2022
Protocol version {3}	version 0.2 on June 7^th^ 15th, 2021, substantial modification n°1
Funding {4}	The French Ministry of Health, DGOS, PHRC IR Interrégional 2020
Author details {5a}	1 Univ. Grenoble Alpes, Inserm, CHU Grenoble Alpes, Grenoble Institut Neurosciences, 38000 Grenoble, France 2 Adult Psychiatry Department CHU Grenoble Alpes 38000 Grenoble, France 3 Early Intervention Psychiatry Department, CH Alpes-Isère, F-38000 Saint-Egrève, France. 4 CERMEP-Imagerie du vivant, Lyon, France. 5 Psychiatry Department, University Hospital Saint-Etienne. INSERM, U1028; CNRS, UMR5292; Lyon Neuroscience Research Center, PSYR2 Team, F-69000, Lyon, France. 6 Centre Hospitalier le Vinatier, F-69500 Bron, France. 7 CHU Saint-Étienne, University Department of Psychiatry and Addiction, 42055 Saint-Étienne Cedex 2, France TAPE Laboratory, EA7423, Jean Monnet University, Saint-Étienne, France 8 SUR-CL3R-PEPS, Centre Hospitalier Le Vinatier, PSYR2 team, Bat 416 – 1st floor; 95 boulevard Pinel, 69678, F-69500, Bron cedex, France. INSERM, U1028; CNRS, UMR5292; Lyon Neuroscience Research Center, PSYR2 Team, F-69000, Lyon, France. Lyon 1 University, F-69000, Villeurbanne, France. 9 Univ. Grenoble Alpes, UMS IRMaGe CHU Grenoble, 38000 Grenoble, France. 10 Psychiatry Department, University Hospital Saint-Etienne, France. 11 Service de Radiologie, CHU de Saint Etienne TAPE EA 7423, Université Jean Monnet, Saint Etienne. 12 Centre Hospitalier Le Vinatier, PSYR2 team, Bat 416 – 1st floor; 95 boulevard Pinel, 69678, F-69500, Bron cedex, France. INSERM, U1028; CNRS, UMR5292; Lyon Neuroscience Research Center, PSYR2 Team, F-69000, Lyon, France. Lyon 1 University, F-69000, Villeurbanne, France. Université Jean Monnet Saint Etienne, F-42000, Saint Etienne, France.
Name and contact information for the trial sponsor {5b}	Julien Colombat, Clinical Project Manager Direction de la Recherche Clinique et de l’Innovation, CHU Grenoble Alpes, DRCI, CS10217, 38043 Grenoble Cedex 9 Tél : +33 4 76 76 56 09 jcolombat@chu-grenoble.fr / accueilrecherche@chu-grenoble.fr
Role of sponsor {5c}	The study sponsor and the funder of the study had no role in the study design, and will not have any role during collection, analyses and interpretation of the data, decision to submit results and writing of study reports.

## Introduction

### Background and rationale {6a}

Schizophrenia is a chronic psychiatric disorder that affects around 1% of the population worldwide. The disorder is recognized as the 8th leading cause of handicap in young adults by the World Health Organization [1]. Life expectancy is reduced by 10–20 years of age, as a consequence of the increased prevalence of non-psychiatric comorbidities, substance abuse, and suicide rates in this population [2]. Schizophrenia has also a profound effect on the patient’s environment, as demonstrated by high levels of subjective burden on relatives of diagnosed individuals [3, 4].

Schizophrenia results from an interaction between vulnerability genes and environmental risk factors. This interaction alters brain development and triggers the clinical manifestations of the disorder in early adulthood. The diagnosis is made primarily on the basis of symptoms, including positive (delusions/hallucinations), negative (flattened affect/avolition), and disorganization (disorganized thoughts/odd behavior) symptoms, associated with reduced psychosocial functioning. At present, treatment mainly consists of antipsychotic medications combined with psychosocial interventions [1]. Treatment-resistance remains an enduring feature for 20–60% of patients [5, 6], which stresses the need to develop further therapeutic approaches.

In parallel to the traditional symptomatology, deficits in cognition (i.e., memory, attention, reasoning, social functioning) contribute significantly to disability and suffering in individuals with schizophrenia. Cognitive deficits have been closely linked to alterations in early auditory processing (EAP) that occur in auditory cortical areas [7–9]. Mechanistically, alterations in EAP have been demonstrated to lead to poor functional outcomes associated with impaired cognition in patients [10–12]. At the molecular level, EAP deficits involve impairments at both cortical and subcortical stages of processing, particularly involving GABAergic, glutamatergic, and N-methyl-D-aspartate receptor (NMDAR)-mediated processes [13]. EAP deficits can be traced using multimodal behavioral and neural measures of EAP, including behavioral tone-matching performance [8], functional neuroimaging of the auditory cortex [14], and early auditory event-related potentials [15, 16] which could thereby serve as surrogate endpoints in procognitive intervention studies in schizophrenia.

Preliminary evidence indicates that cognitive deficits in schizophrenia can be improved with a reliable and safe non-invasive brain stimulation technique called tDCS (transcranial direct current stimulation). tDCS consists of the application of a direct electrical current through two electrodes placed on the scalp to modulate cortical activity. Applied repeatedly over the left frontal and temporal cortices, tDCS significantly improves cognitive performance such as memory and attention in patients with schizophrenia [17, 18]. In parallel to the cognitive improvement, a large amount of randomized sham-controlled studies have reported promising beneficial effects and safety of fronto-temporal tDCS on core clinical manifestations of the disorder such as hallucination and negative symptoms [19–21]. We have further suggested the use of tDCS as a safe first-line agent to both improve the clinical and cognitive manifestations of at-risk mental state and prevent the onset of frank psychosis [22].

Trials are needed to verify the results of cognitive improvements after tDCS in larger schizophrenia cohorts with adequate sham control and randomization. In that sense, additional tDCS trials in schizophrenia are ongoing and receive important funding from governments and foundations across the world [23, 24]. Despite these promising aspects, two areas of uncertainty warrant additional attention. First, meta-analyses of phase III trials indicate that a significant proportion of patients derive no cognitive benefits after tDCS treatment [25, 26]. Second, the neurobiological mechanisms of cognitive changes after tDCS have been poorly explored in trials and are thus still unclear, which hinders the advance of neurostimulation approaches for schizophrenia. There is therefore a critical necessity to develop biomarkers that can help predict which subset of patients will or will not benefit from tDCS, and help determine the biological underpinnings of tDCS-induced cognitive changes.

### Objectives {7}

#### Objective 1 (main objective) and hypothesis

Objective 1 is to compare the efficacy of a left fronto-temporal active tDCS treatment (10 sessions of 20 min delivered over 5 consecutive weekdays) versus sham on global cognitive impairment in patients with recent-onset schizophrenia, at 1-month follow-up. At 1-month we will assess the number of responders, defined as patients demonstrating a cognitive improvement greater than or equal to *Z*=0.5 from baseline on the MATRICS Consensus Cognitive Battery total score (MCCB, a standardized test battery to assess cognitive functions in patients with schizophrenia [27–29].

We hypothesize that there will be significantly more responders in the active group.

#### Secondary objectives and hypotheses

##### Objective 2: clinical efficacy

- Objective 2a is to compare the long-term efficacy of active tDCS versus sham on global cognitive impairment in patients with recent-onset schizophrenia at 3-month follow-up.
- Objective 2b is to compare the efficacy of active tDCS versus sham on different cognitive aspects (processing speed, attention/vigilance, working memory, verbal learning, visual learning, problem-solving, emotional awareness) measured with the MCCB in patients with recent-onset schizophrenia at 1- and 3-month follow-up.
- Objective 2c is to compare the efficacy of active tDCS versus sham on different clinical aspects of schizophrenia (hallucinations, positive, negative, disorganization, depression, grandiosity/excitement, manic symptoms, and subjective experience of cognitive impairment) measured with validated symptom rating scales in patients with recent-onset schizophrenia at the end of the tDCS treatment and at 1- and 3-month follow-up.
- Objective 2d is to compare the efficacy of active tDCS versus sham on the outcome (functioning, quality of life) measured with validated rating scales in patients with recent-onset schizophrenia at the end of the tDCS treatment and at 1- and 3-month follow-up.

We hypothesize that subjects randomly allocated to the active group will demonstrate better improvement from baseline cognitive, symptoms, and functional scores than subjects randomly allocated to the sham group.

##### Objective 3: tolerance

- Objective 3 is to compare the clinical tolerance of active tDCS versus sham measured with adverse effects questionnaires in patients with recent-onset schizophrenia at the end of the tDCS treatment.

We hypothesize that subjects randomly allocated to the active group will demonstrate no significant difference in questionnaire scores than subjects randomly allocated to the sham group.

##### Objective 4: response markers and predictors

- Objective 4a is to assess how differences in EAP mechanisms modulate individual cognitive benefits from active tDCS measured with a range of EAP measures in patients with recent-onset schizophrenia at 1-month follow-up.
- Objective 4b is to evaluate whether there are changes in EAP measures in patients associated with cognitive improvement after active tDCS at 1-month follow-up.

We hypothesize that baseline EAP measures will distinguish between participants who have improved cognitive deficits after active tDCS and those who do not, and that changes in EAP measures after tDCS will be only observed in participants with improved cognitive deficits after active tDCS.

### Trial design {8}

**Fig. 1:**
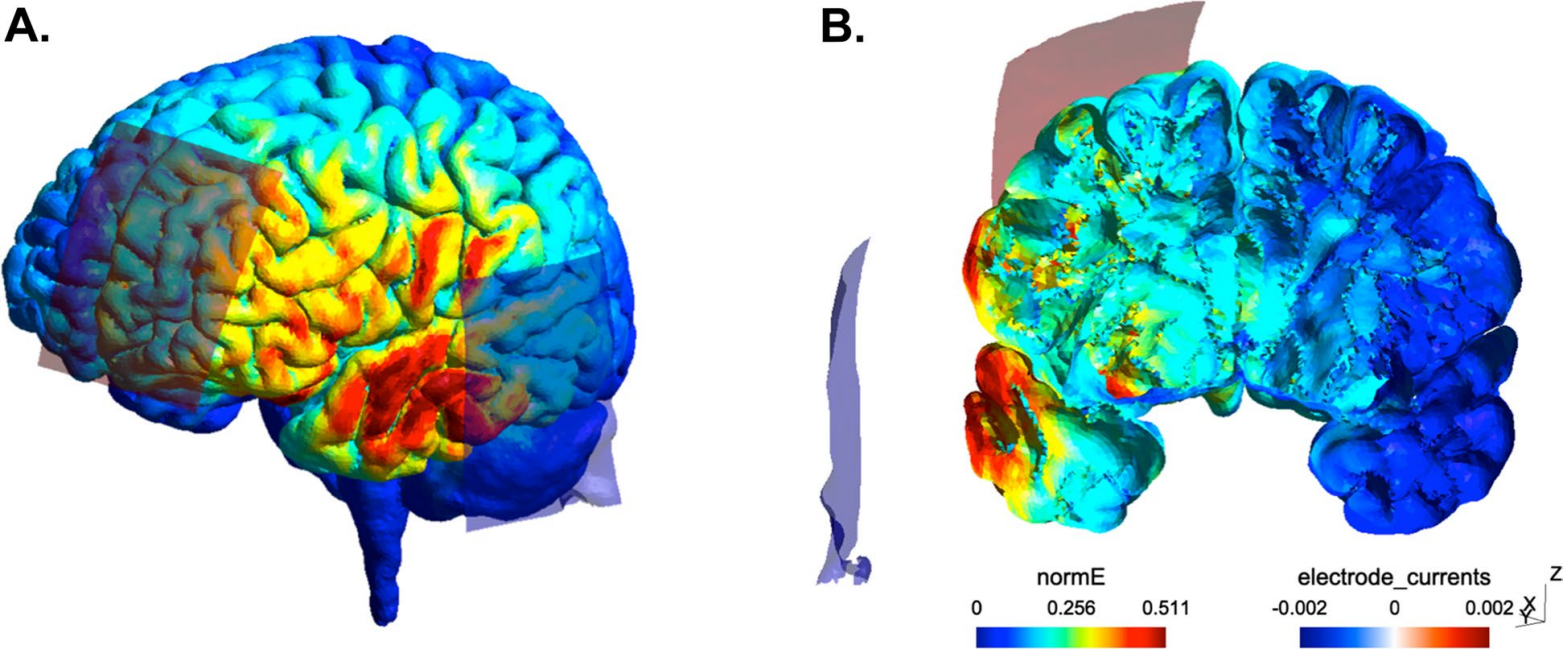
Modelization of current strength pattern associated with the left fronto-temporal tDCS montage (red square: anode, blue square: cathode). **A** Whole brain. **B** Coronal view of the brain; cut through the auditory cortex

**Fig. 2:**
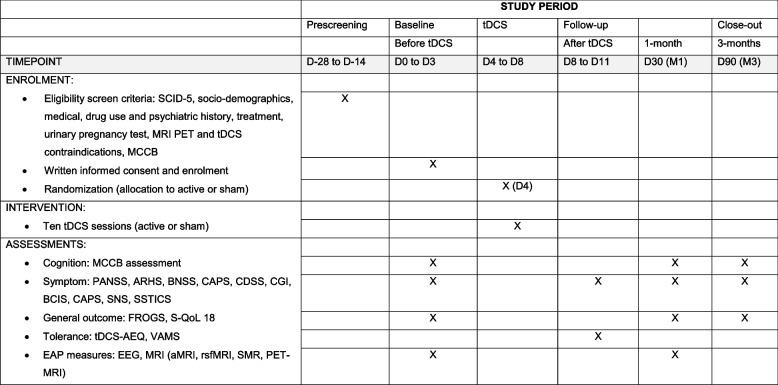
Participant timeline

The present study is designed as a superiority, double-blind, parallel-group, sham-controlled randomized clinical trial with an allocation ratio of 1:1. Study sites will include 4 University Hospital centers in France. In a 1 × 2 factorial design with sham, patients are randomized into two groups: active tDCS and sham tDCS. A total of 60 patients with schizophrenia will be randomly assigned to receive either active tDCS (*N*=30) or sham tDCS (*N*=30). Psychiatric and neuropsychological assessments will be performed at baseline (time of consent), at the end of the tDCS procedure, and at 1 and 3 months following the end of the tDCS procedure (maintenance effect). Auditory biomarker analyses will involve neurobiological measures (multimodal magnetic resonance imaging and neurophysiology) recorded at baseline and at 1 month after tDCS. Study schedule and study flowchart are presented in Figs. 1 and 2.

## Methods: Participants, interventions, and outcomes

### Study setting {9}

Participant recruitment will be conducted across four French centers:

- Centre Hospitalo Universitaire Grenoble Alpes, Department of Adult Psychiatry, Grenoble (10 participants). Neuroimaging platform: IRMaGE.
- Centre Hospitalier Alpes-Isère, Department of Psychiatry, Saint-Egrève (10 participants). Neuroimaging platform: IRMaGE.
- Centre Hospitalier Le Vinatier, Department of Psychiatry, Bron (20 participants). Neuroimaging platform: CERMEP.
- Centre Hospitalo-Universitaire de Saint-Etienne, Department of Psychiatry, Saint-Etienne (20 participants). Neuroimaging platform: IRMAS.

### Eligibility criteria {10}

The inclusion criteria include (1) subjects of both genders, diagnosed with recent-onset schizophrenia (first 3 years of illness), confirmed through the Structured Clinical Interview for the American Psychiatric Association Diagnostic and Statistical Manual of Mental Disorders, 5th edition (SCID-5); (2) aged 18–35 years; (3) intelligence quotient (IQ) > 55; (4) cognitive deficit confirmed by a MCCB total score *T*-score < 40; (5) the subjects should be receiving stable doses of antipsychotics for ≥ 4 weeks; and (6) the subjects are covered by a public health insurance.

The exclusion criteria include (1) pregnant (determined by urine pregnancy test in females of childbearing age) or breastfeeding women; (2) unstable or acute medical conditions; (3) subjects who receive involuntary treatment with no third party available, guardianship or protection of the court; (4) history of cranioencephalic trauma with loss of consciousness or central nervous system diseases that affect the brain; (5) current diagnosis of substance abuse or history of substance dependence in the last 6 months, except nicotine; and (6) MRI, PET or tDCS contraindications.

### Who will take informed consent? {26a}

Informed consent will be obtained by local investigators (psychiatrists) involved in the study. The investigator who obtains the consent will conduct clinical assessments throughout the clinical trial period. Patients will be truthfully and completely informed in comprehensible terms of the requirements, the objectives, the risks, and safety measures. They will be informed of their right to refuse to participate and to withdraw at any time without incurring any penalty or withholding of treatment on the part of her/his psychiatrist and of the investigator, if different. All of this information is described on an information/consent form that will be provided to the participant. Written consent of the participant will be collected by the investigator prior to final inclusion in the study.

### Additional consent provisions for collection and use of participant data and biological specimens {26b}

No additional consent provisions for collection and use of participant data and biological specimens are planned.

## Interventions

### Intervention description {11a}

tDCS (transcranial direct current stimulation subjects) is a noninvasive brain stimulation technique that involves the passage of a small electric current through the scalp and skull to modulate brain activity [10]. The study intervention consists of ten 20-min sessions of active or sham tDCS. Sessions will be delivered twice daily and separated by at least 2 h for 5 consecutive weekdays. The electric current will be generated by an electric stimulator (class IIa medical device). Systems from two commercial distributors are allowed in the study: NeuroConn GmbH (Albert-Einstein-Straße 3, 98693 Ilmenau, Germany, phone: +49 3677 689790; email: info@neuroconn) and Soterix 1x1 tDCS Stimulator (Model 1300A, 8200 Brugge, België, phone +32 50 890 229; email: brunov@vanmed.de).

The procedure for the subject’s installation and tDCS electrode placement will be standardized between study centers using specific training formation sessions. During the entire tDCS session, the subject is at “rest,” comfortably seated in a chair in a quiet room. The subjects will be asked to relax, keep their eyes open, and not perform any particular activity. A clinician will be present for the entire session duration. The current will be applied via a pair of rubber electrodes (7×5 cm, 35 cm^2^) placed on the surface of the scalp according to the international 10/10 system of EEG electrode placement. The anode will be placed equidistant from F3 and FP1 (AF3, left dorsolateral prefrontal cortex, Brodmann areas 8, 9, 10, and 46, depending on the subject). The cathode will be placed equidistant from I7 and P7 (TP7, left auditory cortex, Brodmann areas 22, 41, and 42, depending on the subject). The tDCS montage and current flow modeling are depicted in Fig. 1. The stimulation parameters will be set at 2 mA for 20 min, with a progressive increase during the first 30 s (ramp up) and a progressive decrease during the last 30 s (ramp down) of each session. The impedance of the applied current is monitored by the stimulator during each session. If the limit is exceeded (e.g., increase in impedance due to dryness or electrode drop), the stimulation automatically stops. The cut-off impedance is about 55 kΩ for a 2-mA stimulation. As a safety measure, the impedance will be systematically checked before the start of the stimulation. In case of impedance higher or equal to 55 kΩ, new electrodes will be used.

### Explanation for the choice of comparators {6b}

Sham stimulation is of critical importance in tDCS trials [30]. The sham procedure is developed by the tDCS device manufacturer, which allows using the same tDCS device and the same procedure (i.e., 10 sessions delivered during 5 consecutive weekdays) for both the active and sham procedures. Sham tDCS is based on mimicking typical sensations of active tDCS underneath the electrode site (e.g., itching, tingling). It consists in delivering an active stimulation for a few seconds to mimic these sensations and thus keep participants blind to the intervention. In the sham condition (20-min sessions), the electrodes will be placed in the same positions as in the active group; however, the stimulator will start with a 30-s ramp up, 40-s active stimulation, 18-min and 20-s sham stimulation (i.e., no current), and 30-s ramp down at the end of the stimulation.

### Criteria for discontinuing or modifying allocated interventions {11b}

Modification of the allocated intervention for a given participant (active tDCS or sham tDCS) based upon any reasons will not be allowed. In case of tDCS treatment discontinuation, the participant will be withdrawn from the study.

### Strategies to improve adherence to interventions {11c}

The 10 tDCS sessions will be delivered during the shortest possible interval allowed by safety standards (twice daily during 5 consecutive weekdays). In addition, research staff members will regularly contact the participants by phone to improve retention. These strategies may allow for reducing dropout rates and achieving the primary outcome of the study.

### Relevant concomitant care permitted or prohibited during the trial {11d}

No concomitant care is prohibited during the trial.

### Provisions for post-trial care {30}

After inclusion, no simultaneous participation in other interventional clinical research will be authorized during the trial period. The evaluation of adverse reactions will be carried out for a duration of 3 months.

### Outcomes {12}

All described instruments are validated in the French language.

#### Primary outcome

The primary outcome will be the number of responders at 1 month after tDCS, defined as the proportion of patients demonstrating a cognitive improvement greater than or equal to *Z*=0.5 from baseline on the MATRICS Consensus Cognitive Battery total score (MCCB). The MCCB is a gold-standard standardized test battery to assess cognitive functions in patients with schizophrenia [27, 28]. This criterion has been used and validated in both antipsychotic and cognitive remediation trials in schizophrenia [29, 31]. To avoid learning effects, the MCCB will use different sets of parallel tests with the same difficulty levels at baseline and 1-month.

#### Secondary outcome

##### Secondary outcome 2: clinical efficacy

Outcome 2a will be the number of responders at 3 months.

Outcome 2b will be the changes from baseline to 1-month and 3-month endpoints in each MCCB domains subscores (processing speed, attention/vigilance, working memory, verbal learning, visual learning, problem-solving, emotional awareness) and total score. To avoid learning effects, the MCCB will use different sets of parallel tests with the same difficulty levels at 1 month and 3 months.

Outcome 2c will be the changes from baseline to after tDCS and 1-month and 3-month endpoints in the following symptom measures:

- Schizophrenia symptoms will be assessed using the PANSS (Positive and Negative Syndrome Scale) total score. In addition, PANSS subscores for positive, negative, disorganization, depression, and grandiosity/excitement symptoms will be used as outcomes [32].
- Auditory hallucinations, one of the key symptoms of schizophrenia, will be assessed using the AHRS (Auditory Hallucination Rating Scale) [33].
- Negative symptoms will be additionally assessed using the Brief Negative Symptom Scale (BNSS) [34].
- Depressive symptoms will be assessed using the Calgary Depression Scale for Schizophrenia (CDSS) total score [30].
- Global symptom severity and treatment response will be assessed using the Clinical Global Impressions Scale (CGI) total score [35].
- Cognitive insight abilities will be assessed using the Beck Cognitive Insight Scale total score [36].
- Perceptual anomalies will be assessed using the self-rated Cardiff Anomalous Perceptions Scale (CAPS) total score [37].
- Subjective experience of negative symptoms will be assessed using the self-rated Self-evaluation of Negative Symptoms (SNS) total score [38].
- Subjective experiences of cognitive impairment will be assessed using the self-rated Subjective Scale To Investigate Cognition in Schizophrenia (SSTICS) total score [39].

Outcome 2d will be the changes from baseline to 1-month and 3-month endpoints in the following general outcome measures:

- Functional outcome will be assessed using the FROGS (Functional Remission Observatory Group in Schizophrenia) total score [40]
- Quality of life will be assessed by the Schizophrenia Quality of Life Questionnaire Short Form (S-QoL 18) total score [41]

##### Secondary outcome 3: tolerance

Outcome 3 is the score after the last tDCS session in the following tolerance measures:

- tDCS-AEQ (adverse effects questionnaire) [42]
- VAMS (visual analog mood scale) [43].

##### Secondary outcome 4: response markers and predictors

Outcome 4a is the differences at baseline in the following EAP measures between patients with cognitive improvement and patients without cognitive improvement after active tDCS:

- Correlations (*z*-scores) between left prefrontal and temporal cortical areas (i.e., areas stimulated with tDCS) measured with resting-state functional magnetic resonance imaging (MRI).
- Spectral power (dB) and inter-assay coherence (%) in gamma frequency (40-Hz) during specific auditory paradigms (auditory steady-state, oddball, tone-matching) measured with electroencephalography (EEG).
- GABA and glutamate levels (mM) within left prefrontal and temporal cortical areas measured with resting-state Magnetic Resonance Spectroscopy (MRS)
- radiotracer binding potential on GABA-A receptors (Binding Potential) within left prefrontal and temporal cortical areas measured with resting-state [^11^C]flumazenil positron emission tomography MRI (PET-MRI).

Outcome 4b is the changes between baseline and 1-month follow-up in the same EAP measures in patients with cognitive improvement compared to patients without cognitive improvement after active tDCS.

### Participant timeline {13}

The participant timeline is detailed in Fig. 2 (SPIRIT figure).

### Sample size {14}

Based on previous tDCS studies in patients with schizophrenia (rev. in [17], we assume that a third (33%) of participants in the active tDCS group and 5% of participants in the sham tDCS group will be responders (improvement greater than or equal to *Z*=0.5 from baseline on the MCCB total score). Based on this estimated difference, we calculated that 60 patients are required to demonstrate a difference between the two groups with a power of 80% (*p* = 0.05).

### Recruitment {15}

We developed a multicenter study with four centers in France to achieve sufficient recruitment. Participants with recent-onset schizophrenia will be recruited in each study center with the help of local early-intervention psychiatric teams that specialize in the early stages of schizophrenia. Potential participants will be approached at their regular clinic consultations by investigators with the study information leaflet. All the investigators have already proven their abilities to achieve noninvasive brain stimulation studies (including tDCS) in patients with schizophrenia as highlighted by several international publications. All the investigators are active members or have been trained by members of the French association for the use of noninvasive brain stimulation in psychiatry (see https://www.afpbn.org/sections/step). Annual joint meetings between investigators are scheduled, and periodical newsletters will be sent to all the investigators and research members involved in the study to inform about updates and important points. In case of recruitment issues, new study centers will be open.

## Assignment of interventions: allocation

### Sequence generation {16a}

For the trial, patients will be randomly assigned to either the active tDCS group (n = 30) or the sham tDCS group (*n* = 30) with a 1:1 allocation ratio, using an online randomization system (IWRS Interactive Web Response System). The randomization will be stratified per study center using block randomization. The randomization list will be computer generated by the study sponsor and disclosed to the participants, the investigators and research staff members who enroll participants or conduct interventions.

### Concealment mechanism {16b}

For the trial (30 active, 30 sham), randomization will be conducted using the online IWRS system. The randomization will take place on the day of the first tDCS session in order to avoid the allocation of a sequence that will not be used and to ensure allocation concealment.

### Implementation {16c}

Participants who fulfill the inclusion criteria and give consent will be enrolled by study investigators. Participants will be assigned a unique anonymous identification code by an investigator. This code is composed of the number of the study center number, the participant’s initials (first letter of the name, first letter of the surname), and the last number from 1 to 60. The code will be notified in an electronic Case Report Form (eCRF). Participants will be randomized by an investigator using the online IWRS system, resulting in random 1:1 allocation into one of the 2 study arms (*N*=30 active tDCS and *N*=30 sham tDCS). The IWRS system will provide a 5-digit number code that will be notified at inclusion in the eCRF. The code will be digitally entered into the tDCS device to deliver either to active or sham tDCS.

## Assignment of interventions: blinding

### Who will be blinded {17a}

Blinding will be maintained at 3 levels: participants, research staff members including investigators, and data analysts. If different from the investigator, care providers will be also blinded to the intervention. Blinding for the tDCS condition will be achieved for the research staff members who will administer the tDCS by the use of a randomization code (see details in §16c) and for the participants by ensuring identical appearance and sensation for both active and sham conditions (see details in §6b). The duration of the intervention will be 20 min in both active and sham sessions. Outcome assessments, imaging, and biological data will be collected and analyzed by research staff members blind to group assignment and different from the staff member who will administer the tDCS.

### Procedure for unblinding if needed {17b}

Unblinding will be permissible under the following circumstances during the trial period: appearance of non-inclusion criteria, withdraw of consent to participate in the study, worsening of the clinical condition observed by the investigator justifying the discontinuation of the protocol, and serious adverse effects. Participant’s allocated intervention will be revealed to the subject by the investigator who enrolled the participant.

## Data collection and management

### Plans for assessment and collection of outcomes {18a}

Primary outcome will be assessed by trained neuropsychologists and other outcomes will be assessed by neuropsychologists, psychiatrists, and members of the research staff. All participating investigators and other research staff members that will collect outcomes have been trained for the described assessments. Study subjects will be informed that they can take breaks between tests. Of note, outcomes including neuropsychological tests and clinical and tolerance measurements are frequently used in tDCS and schizophrenia studies. All study sites are experts in those two clinical and research fields. Regarding neurobiological outcomes, all three centers routinely perform electrophysiological and brain imaging examinations. They regularly participate in multicenter trials for which a convergence of acquisition conditions is required. They regularly collaborate with local specialist engineers and manufacturing engineers.

### Psychometric outcomes measures

#### MATRICS Consensus Cognitive Battery total score (MCCB)

The MCCB is a standardized, internationally validated test battery to assess cognitive functions in participants with schizophrenia. The MCCB is a battery of 10 psychometric tests assessing 7 types of cognitive functions. The tests are administered by a neuropsychologist using a "paper/pencil" format or a computerized format. The total duration of the examination is approximately 1 to 2 h. In this study, 3 h are dedicated to each MCCB testing to leave time for breaks between tests.

- Processing speed: *Trail Making Test- Part A; Brief Assessment of Cognition in Schizophrenia- symbol coding subtest; Category fluency test animal naming*
- Attention/vigilance: *Continuous Performance Test- Identical Pairs version.*
- Working memory: *Wechsler Memory Scale, 3rd ed., spatial span subtest; Letter-Number Span test*
- Verbal learning: *Hopkins Verbal Learning Test-Revised*.
- Visual learning: *Brief Visuospatial Memory Test-Revised*.
- Problem-solving: *Neuropsychological Assessment Battery-mazes subtest.*
- Emotional awareness: *Mayer-Salovey-Caruso Emotional Intelligence Test.*

The MCCB is internationally validated, easy to use, sensitive to changes, and is thus used as a reference in clinical trials involving participants with schizophrenia. The total MCCB Z-score is calculated from the weighted average of all MCCB test scores. This score corresponds to the number of standard deviations between the mean value of the patient and the value of a healthy control population (*Z*-score=25.1 ±SD=10) [44]. This score has excellent test-retest and inter-judge reliability (correlation coefficients > 0.90) and is used as a gold-standard index to measure the response of cognitive deficits to treatment in schizophrenia [27, 28]. Cognitive response is defined as an improvement greater than or equal to *Z*=0.5 after treatment. This criterion has a low probability of being related to chance and has been shown to be sufficient for the cognitive improvement to be subjectively felt by the participant [29, 31]. We chose to measure cognitive improvement 1-month after tDCS as most studies show that the effect of tDCS-induced brain modulations on cognitive abilities requires several weeks to be objectively measured [17, 20].

#### Positive and Negative Syndrome Scale (PANSS)

The PANSS is a 30-item clinician-rated scale of the main clinical symptoms observed in schizophrenia. The items are rated on a Likert-type scale, ranking from no symptom (1) to highest intensity (7) of the symptom. The PANSS evaluates the intensity of symptoms based on 3 categories (7 positive items, 7 negative items, and 16 general psychopathology items) or 5 dimensions positive, negative, disorganization, depression, grandiosity/excitement. The PANSS is validated in the French language and has a good consistency and inter-judge reliability (coefficients > 0.60) [32, 45].

#### Auditory Hallucinations Rating Scale (AHRS)

The AHRS is a 7-item clinician-rated scale that generates a detailed description of auditory hallucinations severity for the last 24-h time period. The items are rated on Likert-type scales, ranking from no feature (0) to highest intensity (5, 6, 7, or 9 depending on the individual item) of the auditory hallucination feature. The measured features are frequency, vividness, loudness, length (single words, sentences, phrases, or extended discourse), attentional salience, degree of distress associated with auditory hallucinations, and the number of distinct speaking voices. The AHRS-related “Hallucination Change Scale” (HCS) can be used as outcomes to assess changes from baseline to follow-up in auditory hallucinations severity. The AHRS is validated in the French language and has good test-retest reliability and inter-judge reliability (coefficients > 0.60) [33, 46].

#### Brief Negative Symptom Scale (BNSS)

The BNSS is a 13-item clinician-rated scale specifically developed to measure negative symptoms in participants with schizophrenia for the last week period. The items are rated on a Likert-type scale, ranking from no alteration (0) to highest alteration (6). The measured negative symptom dimensions are blunted affect, alogia, asociality, anhedonia, avolition and associated distress. The BNSS is validated in the French language and has a very good consistency and inter-judge reliability (coefficients > 0.80) [34, 38].

#### Calgary Depression Scale for Schizophrenia (CDSS)

The CDSS is a 9-item clinician-rated scale specifically developed to measure depression symptoms in participants with schizophrenia for the last 2-weeks period. The items are rated on a Likert-type scale, ranking from no symptom (0) to highest intensity (3). The CDSS is validated in the French language and has a good consistency and inter-judge reliability (coefficients > 0.60) [30, 47].

#### Clinical Global Impression (CGI)

The CGI is a brief 3-item clinician-rated scale that involves the assessment of the severity of the disorder at baseline (item 1), the assessment of overall improvement at follow-up (item 2), and the measurement of the therapeutic index which assesses the clinical efficacy and associated side effects (item 3) [35, 48].

#### Beck Cognitive Insight Scale (BCIS)

The BCIS is a 15-item self-questionnaire developed to measure depression cognitive insight in patients with schizophrenia and early stages of psychosis. Each item is rated by the participant on a 4-point Likert scale ranging from 0 (“do not agree at all”) to 3 (“agree completely”). Two cognitive domains are measured: “self-reflectiveness”, which represents the ability to re-evaluate unusual experiences and correct erroneous inference, and “self-certainty,” which evaluates one’s tendency to be overconfident about one's own judgments. The BCIS is validated in the French language and has good internal consistency (coefficients > 0.60) [36, 49, 50].

#### Cardiff Anomalous Perceptions Scale (CAPS)

The CAPS is a 32-item self-rated questionnaire developed to perceptual anomalies in participants with schizophrenia. Each item is presented as a question requiring an answer of “yes” or “no”. The measured perceptual anomalies are Schneiderian first-rank symptoms, temporal lobe experiences, and chemosensations. The CAPS is validated in the French language and has satisfactory psychometric parameters [37, 51].

#### Self-evaluation of Negative Symptoms (SNS)

The SNS is a 20-item self-rated questionnaire specifically developed to assess the subjective experience of negative symptoms of schizophrenia for the last week period. The participant puts a cross in the box next to the response that best corresponds to her/his current feelings based on the previous week, scoring 0 (strongly disagree), 1 (somewhat agree), or 2 (strongly agree). The SNS is validated in the French language and has a good consistency (coefficients > 0.60) and satisfactory acceptance by participants [38].

#### Subjective Scale To Investigate Cognition in Schizophrenia (SSTICS)

The SSTICS is a 21-item self-rated scale that assesses the participant’s subjective experiences of cognitive impairment as indicated from objective tests. The scale consists of 21 Likert-type questions set in the context of everyday activities and situations. The participant puts a cross in the box next to the response that best corresponds to her/his recent feelings, scoring from 0 (never) to 4 (very often). The SSTICS is validated in the French language and has a good consistency (coefficients > 0.60), stability over time, and correlations with objective measures of cognitive abilities [39].

#### Questionnaire of the Functional Remission Observatory Group in Schizophrenia (FROGS)

The FROGS is a 19-item clinical-rated scale that measures the core aspects of functional remission in schizophrenia based on 3 factors (social functioning, daily life, and treatment). The items are rated on a Likert-type scale ranking from does not (1) to does completely (5). The FROGS is validated in the French language [40].

#### Schizophrenia Quality of Life Questionnaire Short Form (S-QoL 18)

The S-QoL18 scale is 18-item self-rated questionnaire measuring eight dimensions of quality of life: psychological wellbeing, self-esteem, family relationships, relationships with friends, resilience, physical well-being, autonomy, and sentimental life. The participant puts a cross in the box next to the response that best corresponds to her/his recent feelings on a 5-point Likert scale. The S-QoL 18 is validated in the French language and has satisfactory psychometric to evaluate the quality of life in participants with schizophrenia [41].

#### tDCS adverse effects questionnaire (tDCS-AEQ)

The tDCS-AEQ is the most frequently used questionnaire to measure the most reported side effects of tDCS (headaches, neck pain, mood alterations, and seizures, rated by the participant on a scale of 0 to 5) [42].

### Neurobiological outcomes measures

#### Electroencephalography

EEG recordings will be carried out using a 64-electrode EEG (BrainVision actiCHamp THE amplifier). Participants will be comfortably seated in a chair in a quiet room. An appropriately sized EEG cap will be placed on the subject’s scalp and a conductive gel will be applied between the scalp and each electrode. At the end of the examination, the electrodes and the EEG cap are removed and the participants will be offered to wash their hair by providing shampoo and towels. The EEG examination lasts approximately 2 h including set-up and breaks. The following auditory tasks will be performed by the participant during the EEG recording:

Auditory Steady-State task (ASSR) will be played through a headset (Sennheiser HD 558) placed on the EEG cap. The ASSR stimuli consist of sound “clicks” of 1 ms duration and 80 dB intensity, broadcast in 500 ms sequences at a frequency of 20Hz, 30Hz, or 40Hz. Three series of 200 stimuli will be broadcast for each frequency [52].

Tone-matching Task (TMT) will be played through the same headset. This computerized task consists in presenting to subjects pairs of non-verbal short basic “beeps” tones (300 ms) with a brief silent interval between tones of each pair (500 ms). Within each pair, tones are either identical or differ in a basic feature (e.g., frequency, length, intensity) by specified amounts. Participants have to respond by pressing “same” or “different” on a 2-button press [8, 53].

Auditory oddball task will consist of sequences of tones presented in random order during a passive (block 1) and an active (block 2) listening condition. Standard stimuli (70% sequential probability) will be harmonic tones composed of three superimposed sinusoids (500, 1000, and 1500 Hz) ~ 80 dB, 100ms in duration with 5-ms rise and fall time. Frequency, intensity, and duration deviants (10% probability each) will be 10% lower in frequency, 10 dB lower in intensity and 50 ms longer in duration respectively. At the beginning of each run, the first 15 auditory stimuli are standards. During passive listening, participants had to simply listen to the tones. During active listening they had to attend and press a key in response to the deviant tones. Passive will elicit an N1 component and mismatch negativity (MMN). Active listening will elicit a P3 component [54, 55].

#### Magnetic resonance imaging

MRI resting-state acquisitions will be carried out using 3-Tesla MRI Scanners. Participants will be positioned on a special table that slides into the MRI. To avoid head movements during the examination, head restraints will be used. The subject will wear a headset designed to attenuate magnet noise while allowing to communicate with the experimenter and notify wish to interrupt the examination at any moment. During all the MRI acquisitions, the subject will be asked to relax, to look at a cross on a screen and not to think about anything. After installation of the participant in the MRI scan, a laser light will be aligned on the orbito-meatal line. This alignment will be regularly checked and corrected if necessary. The MRI examination lasts approximately 2 h including set-up and breaks. The following acquisitions will be carried out:

- aMRI: A few minutes of anatomical MRI sequence (T1-weighted 3D MP-RAGE) will be used for spatial normalization in stereotactic space, anatomical segmentation and parcellation, and extraction of time activity curves by regions during the next acquisitions. Cerebral areas of each participant will be mapped by deformations of standard atlases on the T1 image of the subject.
- rsfMRI: A 15-min resting-state functional MRI sequence will be acquired in axial multislice using traditional gradient echo sequences with a maximum temporal resolution of 3 s. The acquisition will be used to establish maps of the activation and functional connectivity of the fronto-temporal cortical network. A short acquisition to characterize the local inhomogeneities of the B0 magnetic field will be included in order to correct the geometrical distortions they induce.
- MRS: A 30-min acquisition of monovoxel magnetic resonance spectroscopy with GABA editing will be carried out. Two large voxels of interest (left prefrontal and temporal cortices, dimension 3×3×3 cm3 each) will be targeted based on T1 anatomical images. The acquisition will be performed by a "MEGA-PRESS" MRI sequence, which simultaneously removes the water signal and captures the spectral resonance of GABA (γ-CH2, 3 ppm) to estimate its relative concentration (mM) to a stable reference peak. Parameters will be the following: TR/TE = 2000/68 (ms), water signal suppression, 320 excitations divided into 80 dynamics with editing pulse alternately applied at 7.46 and 1.90 ppm between dynamics, phase cycling on 8 excitations per dynamic. Spectra without water signal suppression will also be acquired as a reference for pre-processing.
- PET-MRI: A positron emission tomography acquisition with the GABA marker ([^11^C]flumazenil) will be carried out simultaneously to the MRI sequences described above (PET-MRI). A preliminary interview with a nuclear physician will be performed on the day of the PET-MRI examination. The PET scanner, associated with the MRI imager, is a Biograph mMR, Siemens Healthcare, with an internal tunnel diameter of 60 cm and a useful field of view of 50×50×50 cm^2^ in MRI and 59×59×26 cm^2^ in PET. The PET images produced are volumes of 127 slices of 2mm thickness. The spatial resolution (NEMA standard) is 4.3 mm (total width at half height, FWHM) and isotropic at the center of the field of view. The combination of PET and MRI scans will, after coregistration, improve the spatial resolution and thus refine the data analysis. PET acquisition is performed simultaneously with the MRI sequences described above. PET-MRI requires the installation of a venous catheter by a medical electroradiology manipulator. In our protocol, carbon-11-labeled flumazenil will be synthesized only once per participant. After MRI tracking and attenuation correction sequences have been performed, the injection of the [^11^C]flumazenil 2.5 MBq/kg bolus will be performed at the same time as the start of the PET recording. Dynamic PET data will be recorded 60 min after the injection of [^11^C]flumazenil. Of note, only one of the investigation centers (Centre Hospitalier Le Vinatier) has a PET-MRI device and will thus participate in the PET-MRI acquisition.

### Plans to promote participant retention and complete follow-up {18b}

To promote participant retention, participants will earn 200€ after completing all visits of the trial. To minimize missed study visits and loss to follow-up, all participant visits will be scheduled by a member of the research staff reminded to the participants by phone calls or text messages the day before. The importance of being present at each visit will me reminded to the participant. In case of missed visit, the investigator will seek a new appointment with the participant.

### Data management {19}

In each study site, clinical research assistants will conduct the local study monitoring, which includes appointment management, data recording, and storage in the secured eCRF. A paper version of the CRF will be used to ensure direct ratings during visits and to collect self-questionnaires. Clinical research assistants will continually check the quality of the data, compare the data to medical records and contact the outcome assessors in case of missing or aberrant data. A statement by which all clinicians of the research staff will have direct access to medical records is included in the consent form. Of note, these professionals are bound by strict confidentiality rules and are not allowed to disclose any personal identity or medical information. At specific time points, the quality department of the study sponsor will independently check the consent forms and eCRF for data abnormalities according to standard ranges and, in such cases, inform the investigator. The informed consent form will include a statement by which the patients allow the sponsor’s duly authorized personnel (trial monitoring team) to have direct access to original medical records which supports data on the eCRF (e.g., patient’s medical file, appointment books, and original laboratory records).

### Confidentiality {27}

Both CRF (electronic and paper versions) will be declared to the CNIL (French National Commission for Data Protection and Liberties). Paper CRFs will be kept in locked files at the study sites for 15 years. In all study-related documents, participants will appear only in the form of an ID code (see §16c for details) to ensure confidentiality. All members of the research staff who will have direct access to the data before, during, and after the trial will be bound to strict confidentiality rules and will not be allowed to disclose personal or medical information.

## Statistical methods

### Statistical analysis plan

In general, the normality of the data distributions will be investigated with Shapiro-Wilk tests. Between-group comparisons will be performed with Mann-Whitney *z*-tests and two-sample *t*-tests for non-normal and normal continuous outcomes, respectively. Categorical data will be compared with chi-square tests. Both intention-to-treat and per-protocol analyses will be conducted. In case of significant interactions, post hoc analyses will be conducted using appropriate contrasts. Analyses will be adjusted or stratified by the study site as part of a sensitivity analysis. The significance threshold will be set at *P*-value < 0.05 and appropriate effect sizes of significant results will be calculated.

### Statistical methods for primary and secondary outcomes {20a}

#### Main outcome

The percentage of “responders” showing an improvement in global cognitive improvement (increase ≥ 0.5 of the global MCCB *Z*-score at 1-month follow-up) will be compared between the active tDCS group (*N*=30) and the sham tDCS group (*N*=30) using a chi-square test. In addition, a binomial test will be used to test the superiority of the proportion of responders to active tDCS vs. sham tDCS.

#### Secondary outcomes

##### Secondary outcome 2: clinical efficacy

- Outcome 2a: the percentage of “responders” and the number of responders at 3-month follow-up will be compared using the same tests as for the main outcome.
- Outcomes 2b: the changes from baseline to endpoints in MCCB cognitive domains subscores will be analyzed using mixed effects regression models with group (active or sham) and time (before tDCS, 1-month follow-up, 3-month follow-up) as independent variables.
- Outcomes 2c: the changes from baseline to endpoints in clinical symptom measures will be analyzed using mixed effects regression models with group (active or sham) and time (before tDCS, after tDCS, 1-month follow-up, 3-month follow-up) as independent variables.
- Outcomes 2d: the changes from baseline to endpoints in schizophrenia outcome will be analyzed using mixed effects regression models with group (active or sham) and time (before tDCS, 1-month follow-up, 3-month follow-up) as independent variables.

##### Secondary outcome 3: tolerance

- Outcome 3: tDCS tolerance questionnaire scores measured after tDCS will be compared between the active tDCS and sham tDCS groups using mean comparison tests.

##### Secondary outcome 4: response markers and predictors

- Outcome 4a: the difference at baseline between responders and non-responders in EAP measures will be analyzed using mixed effects regression models with cognitive improvement status (responder or non-responder) and group (active or sham) as independent variables.
- Outcome 4b: the changes from baseline to 1-month follow-up in EAP measures will be analyzed using mixed effects regression models with cognitive improvement status (responder or non-responder), group (active or sham), and time (before tDCS and 1-month follow-up) as independent variables.

### Additional note on preprocessing of neurobiological data (outcomes 4a and 4b}

#### Electroencephalography

The electrophysiological data set will be amplified using the BrainAmp™ acquisition system (Brain Products, Inc.) using a sampling rate of 1 kHz, a resolution of 0.1 μV, and bandpass analog filtering between 0.01 and 500 Hz. A principal component analysis will remove artifactual sources. Temporal periods ranging from −200 ms to 750 ms around the emission of each auditory stimulus will be extracted and normalized to baseline using the standard analysis methods recommended for the MatLab software, version 2017a with relevant toolboxes (EEGLab, ERPLab). Time-frequency analyses by Fourier transform will be conducted with a calculation of inter-trial coherence and average spectral powers over the duration of the per-stimuli time periods. Time domain analyses will be conducted with a calculation of event-related potentials amplitude during the auditory oddball tasks (N1, MMN, P300). The sLORETA source imaging technique (http://www.uzh.ch/keyinst/loreta.htm) will be used to examine the temporal activation of brain regions contributing to the observed event-related potential modulation patterns.

#### Magnetic resonance imaging

The acquired data (images, spectra, and raw data) will be transferred to a server for centralization (Shanoir, https://project.inira.fr/shanoir).

aMRI and rsfMRI: pre-processing including movements realignment, correction of geometrical distortions, “slice timing,” coregistration to the anatomical image, normalization to the reference MNI (Montreal Neurological Institute/International Consortium for Brain Mapping stereotactic space) standard space, spatial smoothing, and additional filters will be performed using the SPM software (Statistical Parametric Mapping https://www.fil.ion.ucl.ac.uk/spm). Then, the correlation rates of the preprocessed signals sensitive to BOLD fluctuations will be regressed both on the whole-brain for whole-brain analyses and at the level of cortical areas stimulated by tDCS (left prefrontal and temporal cortices) for region-of-interest analyses as functional connectivity indices (z-scores) using the SPM CONN toolbox.

MRS: pre-processing of spectroscopy data including ER-filtering of the spectra frequencies, phase dynamics correction (Gabor tool), and the Cadzow procedure for noise reduction (Cadzow procedure) will be conducted using the jMRUI124 software (http://www.jmrui.eu). Then, Glutamine and GABA levels (mM) localized in the cortical areas stimulated by tDCS (left prefrontal and temporal cortices) will be extracted and normalized to a reference metabolite using a recently developed tool specifically for GABA quantification (GABA MRS http://www.gabamrs.com).

PET-MRI: Dynamic PET will be corrected for possible subject motion [56]. Through examination of the PET time-activity curves of the radiotracer [^11^C]flumazenil, changes in extracellular GABA levels will be estimated by calculating the radiotracer binding. Parametric maps of non-displaceable binding potential (BP_ND_) [^11^C]flumazenil will be modeled with the SRTM model using the pons as a reference region (region devoid of GABA-A receptors). Regional BP_ND_ extraction will be performed for the left prefrontal and temporal cortices. Group voxel-to-voxel statistical analyses will be performed in the MNI space with the SPM toolbox.

### Interim analyses {21b}

The study will have no interim analysis.

### Methods for additional analyses (e.g., subgroup analyses) {20b}

Subgroup analyses will be performed regarding possible confounders such as the study site. In case of baseline differences between the 2 groups (active tDCS and sham tDCS), statistical analyses will be adjusted to take into account specific variables that may have influenced the results (e.g., antipsychotic dosage, illness duration, and age).

### Methods in analysis to handle protocol non-adherence and any statistical methods to handle missing data {20c}

Protocol non-adherence will be described as follows: characteristics at inclusion, arm (active tDCS or sham tDCS), withdrawal date and visit number, reason for withdrawing, and last outcomes carried forward. For the trial (*N*=60), the analyses will be conducted on the intention-to-treat set, including all randomized participants with at least one assessment conducted after the tDCS treatment (10 sessions). Per protocol analyses will be performed using the whole sample (*N*=60). The last observation carried forward will be used for missing data.

### Plans to give access to the full protocol, participant level-data and statistical code (31c}

The present document describes the full protocol. Statistical codes and anonymized participant-level data will be available from the principal investigator upon reasonable request.

## Oversight and monitoring

### Composition of the coordinating center and trial steering committee {5d}

The steering committee is composed as follows:

- Principal investigator: Dr. Clément Dondé
- Methodology:
  - Scientific advisory: Dr. Julien Bastin, Dr. Jérôme Brunelin
  - Trial methodology and study coordination: Dr. Clément Dondé, Prof. Mircea Polosan
  - Statistical analyses: Dr. Julien Bastin
- Quality control and study safety monitoring: Direction de la Recherche Clinique et de l’Innovation, CHU Grenoble Alpes, DRCI, CS10217, 38043 Grenoble Cedex 9
- Imaging: Dr. Laurent Lamalle, Dr. Inès Troprès, Dr. Nicolas Costes, Dr. Julien Bastin
- Data management: Mrs. Blandine Chanteloup

### Composition of the data monitoring committee, its role and reporting structure {21a}

Data monitoring reports will be ensured by an independent committee mandated by the principal study site (CHU Grenoble Alpes). The members of this committee have no relationship with the investigators and no competing interest related to the study. The initial committee meeting will take place prior to the start of the trial. The committee will meet at least once a year to assess the trial's progress and safety. Prior to each meeting, no data analysis will be performed. If necessary, the trial expansion will be discussed at the final committee meeting. Throughout the trial period, at least annual onsite monitoring visits will be planned to verify the accuracy and quality of the data.

### Adverse event reporting and harms {22}

Safety assessment will be conducted according to the requirements of the MDR 2017/745 and MDCG 2020-10/1 statements (European commission) and article of the R1123-46 (French Public Health Code). The investigator will document in the CRF any adverse event observed of reported by the participant. The causal link with the use of the medical device (tDCS) will be assessed. Any adverse event will be monitored until it is completely resolved.

### Frequency and plans for auditing trial conduct {23}

The principal investigator agrees to allow direct access to the study records to independent regulatory authorities for audit or inspection.

### Plans for communicating important protocol amendments to relevant parties (e.g., trial participants, ethical committees) {25}

Important protocol modifications will require a formal amendment to be approved by the IRB. The principal investigator will be in charge of communicating protocol modifications to relevant parties (principal study site, IRB, trial registry https://clinicaltrials.gov/) and, if approved, to participating sites.

### Dissemination plans {31a}

Peer-reviewed publications and communications of the study results at conferences and congresses are planned. The first article will be based on the primary outcome of the trial. The results from secondary outcomes could be published in separate articles.

## Discussion

The present study is a double-blind, sham-controlled, parallel-group trial designed to investigate the efficacy and tolerance of left fronto-temporal transcranial direct current stimulation (tDCS) as a treatment of cognitive impairment in a multicenter sample of patients with recent-onset schizophrenia. Given the causal role of cognitive defect in daily functioning [57, 58], the utilization of safe and potentially effective neuroenhancement tools such as tDCS for the rehabilitation of cognitive impairment in the early stages of schizophrenia may help to improve the outcomes of the disorder. As the contribution of EAP deficits to cognitive and functional outcomes is increasingly demonstrated [10–12], we will evaluate for the first time the impact of targeting the auditory cortical areas (instead of the temporo-parietal junction) on cognitive response. While several RCTs have already been conducted to test the efficacy of tDCS on cognitive impairment in schizophrenia, most of them were limited by the lack of maintenance effects assessment and small sample size [17]. Here, besides proposing a new fronto-temporal tDCS protocol by targeting the auditory cortical areas, we aim to conduct an RCT with follow-up assessments up to 3 months.

In addition, this study will allow identifying and assessing the value of a wide range of neurobiological EAP measures for predicting and explaining cognitive deficits improvement after tDCS. Alterations in EAP have been demonstrated to lead to poor functional outcomes associated with impaired cognition in patients [10–12], which makes EAP a relevant biomarker for cognitive response. The search of predictive and explanatory biomarkers of clinical response to tDCS is an expanding domain with the potential to improve therapeutic plans offered to patients with schizophrenia, while improving our understanding of underlying tDCS mechanisms of effect on cognitive deficits. The results of this study will streamline participant selection in subsequent tDCS trials, thereby enhancing treatment outcomes and reducing costs through a precision medicine approach. Limitations of this trial include the time commitment needed from participants and investigators, stringent exclusion criteria, risk of dropout during the study, and the possibility that the entire sample will not meet the cut-off for cognitive response.

Overall, if the findings from the present trial confirm our hypotheses, they may contribute to the value of tDCS for the treatment of cognitive impairment in recent-onset schizophrenia. As some subtle disabling cognitive impairments are considered as core features of the pre-clinical vulnerability of schizophrenia, we also hypothesize that fronto-temporal tDCS would be clinically relevant in individuals with an at-risk mental state for psychosis and may constitute a preventive intervention against psychotic onset in the future [22].

## Trial status

The Protocol Version is 1.0 (15^th^ December 2021). The trial is currently ongoing. Recruitment will begin in the winter of 2022 and is expected to finish in 2025.

## Supplementary Information

**Additional file 1.** Ethical approval.

## Data Availability

All members of the steering committee will have full access to the final dataset. The data will be available from the principal investigator to other study investigators or scientists upon reasonable request.

## Abbreviations

AE: Adverse event
AHRS: Auditory Hallucination Rating Scale
BCIS: Beck Cognitive Insight Scale
BNSS: Brief Negative Symptom Scale
BOLD: Blood oxygen level-dependent
CAPS: Cardiff Anomalous Perceptions Scale
CDSS: Calgary Depression Scale for Schizophrenia
CGI: Clinical Global Impressions
CRF: Case report form
DSM: Diagnostic and Statistical Manual of Mental Disorders
eCRF: Electronic case report form
FAR: Facial affect recognition
fMRI: Functional magnetic resonance imaging
HCS: Hallucination changes scores
tDNS: transcranial direct noise stimulation
MRI: Magnetic resonance imaging
PANSS: Positive and Negative Syndrome Scale
RCT: Randomized controlled trial
SAE: Serious adverse event
SNS: Self-evaluation of Negative Symptoms
S-QoL18: Schizophrenia Quality of Life Questionnaire Short Form
tDCS: Transcranial direct current stimulation
VAS: Visual analog scale
