# Supplementary material for: Efficacy and auditory biomarker analysis of fronto-temporal transcranial direct current stimulation (tDCS) in targeting cognitive impairment associated with recent-onset schizophrenia: study protocol for a multicenter randomized double-blind sham-controlled trial
Source: Trials. 2023 Feb 24;24:141. doi: 10.1186/s13063-023-07160-z (PMC9951427; doi:10.1186/s13063-023-07160-z)
Supplement: Supplementary file 1 — Additional file 1. Ethical approval. [file 13063_2023_7160_MOESM1_ESM.docx]

**SUPPLEMENTARY MATERIAL**

**Ethics approval**

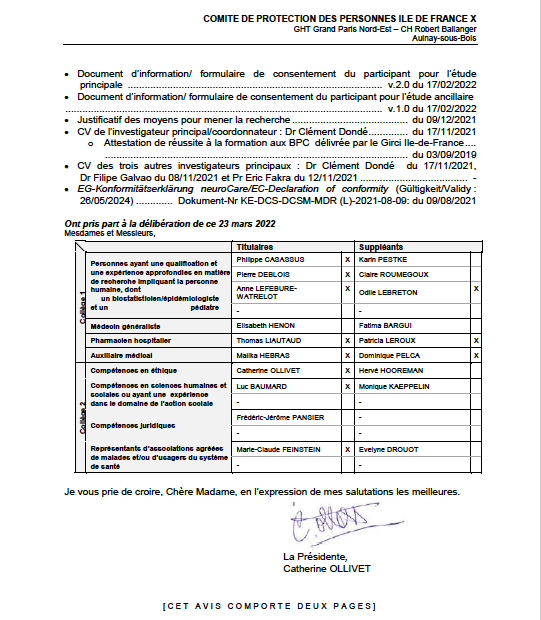

**Ethics approval – english translation of relevant sections**


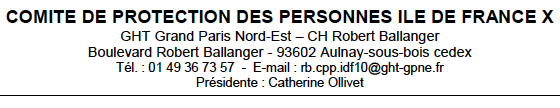


Dear Madam,

The Committee for the Protection of Persons Ile-de-France X has been seized by you on the SI-RIPH2G of a request for an initial opinion for the interventional research referenced above.

The Committee meeting this Wednesday, March 23, 2022 examined the changes made by the sponsor in response to the comments it had made in its opinion of February 15, 2022, and decided to issue a DEFINITIVE FAVORABLE OPINION for the implementation of this study in France.

Please believe, Dear Madam, in the expression of my best regards.

The president, Catherine OLLIVET


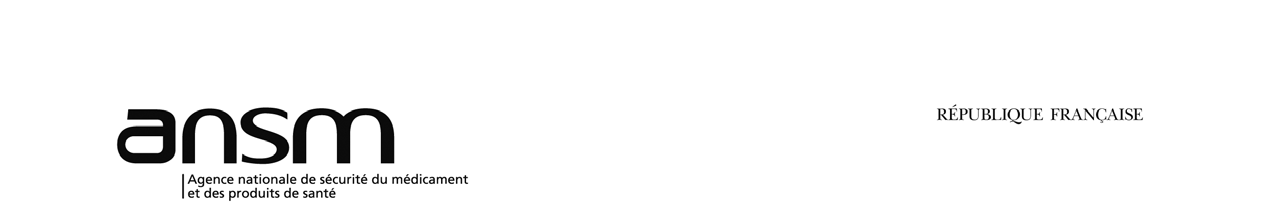


Subject: AUTHORIZATION OF A CLINICAL TRIAL NOT CONCERNING A HEALTH PRODUCT Dear, By e-mail dated January 13, 2022, you sent a request for clinical trial authorization not relating to a product mentioned in article L. 5311-1 of the CSP (Test-HPS):

Having regard to the Public Health Code and in particular Article L. 1123-8 and the regulatory provisions taken for its application, The authorization mentioned in article L. 1123-8 of the public health code is granted for the clinical trial mentioned in object. This authorization is valid for the entire duration of the trial from the date hereof. However, this authorization lapses if the research has not started within the period set by regulation.

Please accept, Madam, Sir, the assurance of my highest consideration.

Gaelle GUYADER Director Authorizations Directorate

**Funding**

**
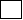
Funding – english translation of relevant sections**

AO DGOS 2020

CHUGA CAMPAIGN RESULTS

Dear, We are delighted to announce that 5 projects led by the CHUGA have been selected for funding at the AOs DGS 2020:

Call type of offer Project title Investigator Director

PRME SOCRATES: Medico-economic evaluation of a strategy with scanner coronary in first line compared to the strategy with functional test in the first line, in patients at intermediate risk of presenting stable coronary artery disease: a prospective, controlled, clinical trial randomized.

**Gilles BARONE-ROCHETTE**

PREPS FIAQLS: Automated indicator feedback for improving the quality of the liaison letter at discharge: a randomized controlled trial in clusters. Bastien BOUSSAT

PREPS COM-ARM: Effectiveness of a communication training program to improve the handling of calls for suspected cardiac arrest at SAMU-centre 15. Multicenter randomized controlled trial step-wedge implementation.

**Guillaume DEBATY**

PHRC-I STICOG: Efficacy and auditory biomarker analysis of fronto-temporal transcranial direct current stimulation (tDCS) in targeting cognitive impairment associated with recent-onset schizophrenia: study protocol for a multicentric randomized double-blind sham-controlled trial. – 250 k€

**Clément DONDÉ**

PHRC-I CLOFISPERM: Study of the efficacy of clofilium on obtaining a pregnancy in intrauterine insemination

**Pascale HOFFMANN**

We would like to congratulate and thank everyone involved in the success of this AO DGOS campaign. 2020:

- Investigative teams
- Methodologists, assembly managers, biostatisticians, regulatory and vigilance units
- Research support structures (Biology, Pharmacy, Imaging and Irmage)

We remain at your disposal for any further information,

Best regards,

Home Research Unit

Ilhem EL AMRANI Tél. +33 (0)4 76 76 74 09

Tiphaine MONTAGNON Tel : +33 (0)4 76 76 67 97

Anastasia ESCHEVINS Tél. +33 (0)4 76 76 79 00

Cyrielle CLAPE Tél. +33 (0)4 76 76 79 00

Contact : [accueilrecherche@chu-grenoble.fr](mailto:accueilrecherche@chu-grenoble.fr)
